# Households forgoing healthcare as a measure of financial risk protection: an application to Liberia

**DOI:** 10.1186/s12939-019-1095-y

**Published:** 2019-12-10

**Authors:** Jacopo Gabani, Lorna Guinness

**Affiliations:** 1 Centre for Health Economics, University of York, York, UK; 2 London School of Hygiene & Tropical Medicine, London, UK

**Keywords:** Health financing, Equity, Liberia, Impoverishment, Catastrophic health expenditure, Forgoing healthcare, Financial risk protection

## Abstract

**Introduction:**

Access to Liberia’s health system is reliant on out-of-pocket (OOP) health expenditures which may prevent people from seeking care or result in catastrophic health expenditure (CHE). CHE and impoverishment due to OOP, which are used by the World Bank and World Health Organization as the sole measures of financial risk protection, are limited: they do not consider households who, following a health shock, do not incur expenditure because they cannot access the healthcare services they need (i.e., households forgoing healthcare (HFH) services). This paper attempts to overcome this limitation and improve financial risk protection by measuring HFH incidence and comparing it with CHE standard measures using household survey data from Liberia.

**Methods:**

Data from the Liberia Household Income and Expenditure Survey 2014 were analysed. An OOP health expenditure is catastrophic when it exceeds a total or non-food household expenditure threshold. A CHE incidence curve, representing CHE incidence at different thresholds, was developed. To overcome CHE limitations, an HFH incidence measure was developed based on CHE, OOP and health shocks data: households incurring health shocks and having negligible OOP were considered to have forgone healthcare. HFH incidence was compared with standard CHE measures.

**Results:**

CHE incidence and intensity levels depend on the threshold used. Using a 30% non-food expenditure threshold, CHE incidence is 2.1% (95% CI: 1.7–2.5%) and CHE intensity is 37.4% (95% CI: 22.7–52.0%). CHE incidence is approximately in line with other countries, while CHE intensity is higher than in other countries. CHE pushed 1.6% of households below the food poverty line in 2014. c approximately 4 times higher than CHE (8.0, 95% CI, 7.2–8.9%).

**Conclusion:**

Lack of financial risk protection is a significant problem in Liberia and it may be underestimated by CHE: this study confirms that HFH incidence can complement CHE measures in providing a complete picture of financial risk protection and demonstrates a simple method that includes measures of healthcare forgone as part of standard CHE analyses. This paper provides a new methodology to measure HFH incidence and highlights the need to consider healthcare forgone in analyses of financial risk protection, as well as the need for further development of these measures.

## Key messages

- Catastrophic Health Expenditures (CHE) and impoverishment effect of out-of-pocket (OOP) health expenditure do not fully represent financial risk protection as people forgoing healthcare are excluded from those measures
- CHE are incurred by 0.4 to 2.1% of households in Liberia, and they are more concentrated in poorer households than in richer households, and 1.6% of households have been pushed into poverty in 2014 by OOP health expenditure in Liberia
- However, an even greater number of households are estimated to forgo healthcare in Liberia (8.0%)
- Omitting a measure of households forgoing healthcare from financial risk protection measures under-estimates the full extent of financial barriers to healthcare on poorer households in Liberia
- Research on households forgoing healthcare should be included in standard financial risk protection measurement to capture the potential gains from universal healthcare coverage.

## Introduction

Universal Health Coverage (UHC) is defined as “ensuring that all people have access to needed healthcare services of sufficient quality to be effective while also ensuring that the use of these services does not expose the user to financial hardship” [40]. Across the globe, UHC and countries’ progress towards it have received increased attention since its inclusion in the Sustainable Development Goals [28]. However, measures of progress towards UHC are complex as they need to consider both the degree of financial risk protection [23] that is achieved as well as changes in service coverage [5]. The global UHC framework for monitoring progress developed by the World Bank and WHO captures these concepts through a multi-dimensional framework, taking into account the services that are covered, who is covered and the degree to which the population access pooled funds and achieve financial protection [5, 32]. In examining financial protection, this includes two indicators: catastrophic health expenditure (CHE) and the impoverishing effect of out-of-pocket (OOP) health expenditure, both of which should be presented in the aggregate and broken down by socioeconomic group in order to capture the distributional impact of healthcare spending [5, 23].

CHE are said to occur when out-of-pocket expenditures as a share of household resources surpasses a given threshold [20, 31]. The thresholds used in CHE analysis are designed to reflect a household’s capacity to pay which can be defined by household income, consumption expenditure or non-food consumption expenditure, depending on the data available and the analyst’s approach [18, 20, 41]. In the case of the impoverishing effect of OOP health expenditure, the threshold describes a poverty line defined at a national or international level [20, 31]. In both cases, the aggregate form addresses the issue of horizontal equity (i.e. people with similar income contribute in similar ways to health system financing [22]); whereas the distribution by socioeconomic group helps us understand the degree to which vertical equity (i.e. wealthier people contribute more than poorer people to health system financing [22]) is being addressed or may need to be addressed.

These indicators have succeeded in providing a useful starting point for measuring progress related to financial protection under UHC. Yet both measures suffer from limitations in their ability to capture the full impact of healthcare need on household resources. The arbitrariness related to choosing the threshold makes it difficult to compare across different analyses [11, 27]). Hsu et al. [11] have addressed this by using a “CHE incidence curve” which can provide meaningful cross-country comparisons [11]. A further criticism of CHE and the impoverishing effect of OOP as measures of progress in financial protection is that they only consider the effects of an *expenditure* [7, 18]*;* households who suffer financial hardship because they cannot afford, or access, healthcare would not be counted. Therefore, CHE and impoverishing expenditure are likely to under-estimate the number of people without financial risk protection. This potential for under-estimation is a problem in countries with high inequality or a large proportion of the population living below the poverty line [21].

**Fig. 1:**
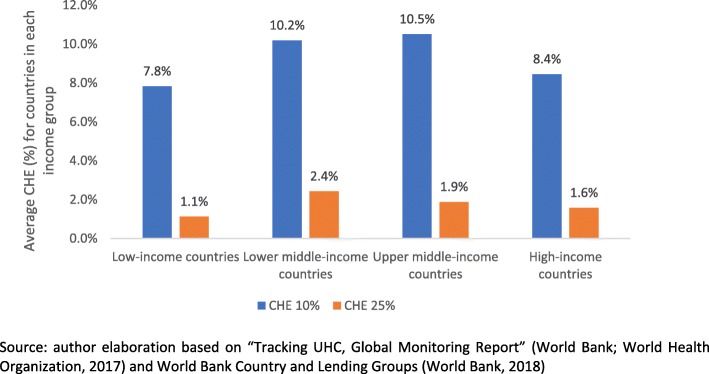
Average Catastrophic Health Expenditure (CHE) of countries depending on their income groups. Source: author elaboration based on “Tracking UHC, Global Monitoring Report” [32] and World Bank Country and Lending Groups [38]

As yet, measures for identifying and counting these missing populations are not used in measuring financial risk protection [17] and monitoring UHC [1], leading to unexpected results regarding financial risk protection. According to the “Tracking UHC, Global Monitoring Report” [32], low-income countries populations are *more* financially protected than middle- and high-income countries populations; such counter-intuitive findings may be driven by the under-estimation of financial risk protection that is implied in CHE and impoverishment measures (Fig. 1).

Liberia is a low-income country where years of conflict have damaged the health system [12]. In 2015, its GDP per capita was the 7th lowest globally, approximately 35% below the average for low-income countries [33]. Health status measures also showed a challenging situation: Liberia’s maternal mortality rate and under-5 mortality rate were respectively 11th and 26th highest globally, approximately 40 and 5% higher than the average for low-income countries [34, 35]. Out-of-pocket health expenditures in Liberia make up 19.6% of total health expenditure in 2015 [39], the percentage of people living below the poverty line is high (50.9% in 2016) [15] and there is no recent study of CHE or impoverishing health expenditure. Access to publicly funded services is open to all citizens, and public healthcare services are financed through domestic revenues. However, the Liberian health system is highly reliant on external financing, as 71% of its total health expenditure is external. Only 7% of total health expenditure is financed by domestic revenues. The remainder is private health expenditure, which is largely out-of-pocket [39]. This paper explores financial risk associated with healthcare in Liberia and proposes a method to complement financial risk protection measures in the WHO/World Bank global monitoring framework for UHC. Alongside standard catastrophic and impoverishing expenditure analyses, it measures how many households are forgoing healthcare services.

## Methods

### Study sample and data

Liberia is a low-income country in sub-Saharan Africa with 4.7 million people, life expectancy of 63 years, GNI per capita of 620 US$ [37] and 0.08 physicians per 1000 people [39]. Data analysed in this paper are taken from the Liberia Household Income and Expenditure Survey 2014–2015 (HIES) [15], the first household income and expenditure survey in Liberia since 1964 [15]. Although the Ebola virus (EVD) hit Liberia in 2014 and interrupted the HIES data collection, approximately 50% of the sample was surveyed so that the data are still representative at the national level [15]. Data required for the analysis of financial risk protection using CHE and impoverishment indicators include OOP, household consumption expenditure, household capacity to pay and the national poverty line. In addition, asset indices are required to explore the distribution of CHE across income quintiles. Table 1 provides a list of variables used in the analyses as well as their definitions and sources.

**Table 1:** List of variables, and their definitions, used in the analysis of Catastrophic Health Expenditure and Healthcare Foregone

#	Variable	Definition	Related Questions/ Database items	Connection with other measures:	Calculation	Source
1	OOP Health Expenditure	All OOP health expenditure in last 12 months	Q10, 11, 15, 16, 20, 22 (section D)	CHE numerator	Sum of Q10, 11, 15, 16, 20, 22	[15]
2	Total Consumption Expenditure	Food and non-food consumption per household in last 12 months	Worksheet HH consumption, item hhtexp	CHE denominator	No calculation, taken directly from database	
3	Non-food consumption expenditure	Non-food consumption per household in last 12 months	Worksheet HH consumption, item nfdtexp	CHE denominator	No calculation, taken directly from database	
4	Capacity to pay	Same as non-food consumption expenditure (#3)				
5	CHE threshold	Threshold beyond which an OOP health expenditure is considered a CHE	n/a		Arbitrary values taken from literature	[31, 32, 41]
6	CHE Incidence	Households incurring CHE, out of total households	n/a	OOP health expenditure (numerator), Consumption expenditure (denominator) and threshold	1 if #1 divided by #2 or #3 is beyond #5 0 otherwise	[6, 9, 20, 29, 31, 41]
7	CHE Intensity	Average OOP health expenditure value beyond CHE threshold (for households incurring CHE)	n/a	OOP health expenditure (numerator), Consumption expenditure (denominator) and threshold	If #6 is 1: #1 divided by #2 or #3 minus #5 0 otherwise	
8	Impoverishment	Households incurring CHE which were pushed below poverty line by CHE	n/a	CHE	1 if total expenditure minus OOP health expenditure is inferior to household’s poverty line, 0 otherwise	[7, 20, 31]
9	Poverty line	Consumption expenditure per household assumed to be a minimum living standard (food/non-food)	n/a	Impoverishment	Sum of adult equivalents in household, times poverty line per adult equivalent (L$65438)	[15]

### Data analysis

#### Catastrophic health expenditure incidence and intensity

OOP health expenditure was defined as the total household expenditure on formal and informal healthcare over a one-year period, excluding health insurance contributions. Formal healthcare is defined modern healthcare services provided by regulated facilities (i.e. government facilities, private facilities and NGO-run facilities), while informal healthcare refers to services provided by unlicensed traditional and faith health [26]. The CHE incidence is the number of people whose OOP exceeds the identified threshold in the given period [20, 31]. Following the World Bank and World Health Organization [32], OOP were compared with total consumption expenditure, and the standard CHE threshold values varied between 10 and 25%. Capacity to pay (i.e. total non-food consumption expenditure) was also used as a comparator and the thresholds varied between 30 and 40% [29, 41]. See Appendix 1 for full details of the methods. Given that no “right or wrong” threshold can be defined [11, 20], a sensitivity analysis was performed to show how CHE incidence varies across the CHE threshold values. The sensitivity analysis is graphically presented by plotting CHE incidence curves against the different threshold values [11].

CHE incidence is also not able to capture *how much the threshold is exceeded by OOP payments.* CHE intensity or overshoot measures *how much* households incurring CHE are affected by CHE [7, 20, 31]. The overshoot for each household was estimated as the difference between the threshold value and the out of pocket expenditure as a proportion of capacity to pay. The average intensity for the population was then estimated as the mean overshoot (see Appendix 2 for full details of the methods).

#### Equity and CHE

A concentration index (CI) was used to measure the level of equity in the sampled population. A CI is a standard “measure that quantifies the degree of socioeconomic-related inequality in a health variable” [20] and has been often used to quantify CHE inequality [3, 7, 9]. The concentration index for CHE incidence *C*_*HC*_ is equal to zero when CHE incidence is distributed perfectly equally (with regards to income), negative if CHE is concentrated among poor households and positive if CHE is concentrated among rich households. A weighted concentration index WHC was then calculated to take account of the distribution of CHE incidence across different socio-economic quintiles. If the concentration index *C*_*HC*_ is negative, then the rank-weighted CHE incidence $\overline{WHC}$ would be greater than the unweighted CHE incidence $\overline{HC}$, reflecting the fact that poorer people are more affected by CHE than richer people (see Appendix 3).

#### OOP health expenditure impoverishment effect

A limitation of comparing total consumption expenditure to poverty lines is that total consumption expenditure includes expenditures, such as health expenditures, that *prevent welfare deterioration rather than increase welfare* [16]*.* For this reason, the impoverishment effect was used to measure how many households were pushed below the poverty line by OOP health expenditures, whether they are catastrophic or not. To measure how many households were pushed below the poverty line by OOP health expenditure, the incidence of poverty before OOP health expenditures was compared with the incidence of poverty after OOP health expenditures [20, 30, 31]. The poverty line was defined as 65,438 L$ - the Liberian poverty line per each adult equivalence, as per the HIES 2014 methodological appendix [15]. The impoverishing effect of OOP health expenditure is the difference between poverty incidence before OOP health expenditure and poverty incidence after OOP health expenditure (see Appendix 4 for the full methods).

#### Households forgoing healthcare

Only households *who have purchased healthcare services can incur CHE*: when the required healthcare services are unaffordable or inaccessible for any reason, a household forgoes healthcare services needed, does not spend any money on healthcare services, and bears the lower quality of life due to the untreated health shock. Such untreated health shock may lead to household impoverishment through loss of productivity and/or income.

Given the above limitation of CHE, HFH incidence has been frequently assessed by including questions on HFH in a survey [17], (e.g. Commonwealth Fund International Health Policy Survey [24], population-based and country specific surveys [4, 10]). This HFH measurement methodology is subjective and is therefore difficult to use in cross-country comparisons. Finally, this method is dependent on having an HFH question in the survey: while limited, there is a cost related to adding a question to a survey.

A needs-based approach such as proposed by Pradhan and Prescott [21] could address this issue of subjectivity [21]. However, their approach appears to be complex and has not been widely applied. This paper develops a simplified needs-based approach to measure HFH incidence. Its limitations are that.

To identify those households that forgo healthcare services, their OOP needs to be below the expected value of the healthcare services that they need. The incidence of households forgoing healthcare services (HFH) could then be represented by the percentage of households that experienced a health shock, have not incurred CHE *and have not spent more than a specific threshold value, in OOP health expenditure.* The threshold value can be chosen to reflect the minimum OOP health expenditure needed to cope with a health shock, for example, the value of a basic healthcare intervention in the country e.g. a blood test. It is important that households forgoing healthcare have not incurred CHE so that the two measures are mutually exclusive and can be summed to estimate the percentage of households who are not financially protected.

The Liberia HIES 2014 reports if a household has had a health shock, where a health shock is defined as the occurrence of at least one event among severe illness, chronic illness, accident or death of a household member [14] within the last 12 months. For the case of Liberia, US$10 threshold was chosen as the minimum value of care needed to cope with a health shock, although such care is likely to cost more than US$10 (as mentioned, a basic blood test, possibly not available in public clinics, may cost US$10 in a private clinic, even without considering transport costs to access state funded healthcare services). Therefore, a household was defined as forgoing healthcare if they fulfilled three criteria: reported a health shock, had a health expenditure of less than US$10 and did not incur CHE (Appendix 5 describes the full methodology).

A sensitivity analysis was then carried out to check how the index varies in relation to the threshold cost of healthcare used (US$10) (thresholds ranging from 0 US$ to 10,000 US$), following the same principles as the sensitivity analysis applied to the CHE estimation.

Sampling weights have been used throughout the analysis to improve sample representativeness [25]. Data were analysed using Microsoft Excel (Microsoft, Washington, USA) and Stata/IC 14 (StataCorp, Texas, USA) for Windows.

## Results

### Descriptive statistics

The characteristics of the sample are presented in Table 2. Most households (60%) lived in urban areas, while the remaining households (40%) lived in rural areas. Households with less than 5 members were 58% of the total, and while in most households (55%) there was a child below 5 years old, there was an adult above 60 years old in only 14% of the households. Almost half (43%) of the heads of household in the sample have no formal education. Most heads of household are male (71%), aged below 44 years old (63%). Approximately one fifth (19%) of all households suffered a health shock in the last 12 months.

**Table 2:** Descriptive statistics: sample characteristics

	% of households	Number of Households in sample (n)
Residence		
Rural	40%	2546
Urban	60%	1539
Household size		
1	12%	443
2	13%	478
3	16%	610
4	17%	704
5+	42%	1850
Household age composition		
Presence of children < 5 years	55%	2353
Presence of adults > 60 years	14%	715
Age of head of household		
< 25 years old	9%	291
25–34 years old	27%	1017
35–44 years old	27%	1138
45–54 years old	20%	848
> 54 years old	17%	791
Education of head of household		
None	43%	1905
Some or completed primary	12%	609
Some or completed secondary	38%	1399
More than secondary	7%	172
Gender of head of household		
Male	71%	3014
Female	29%	1071
Suffered a health shock (chronic or severe illness, accident, or death)		
Yes	19%	765
No	81%	3320

Source: Liberia Household income and expenditure survey, 2014

Relevant expenditure measures are shown in Table 3. The mean annual total household expenditure and OOP health expenditure were L$217,800 and L$2800 respectively (exchange rate: US$ 1 = 92 L$, source: oanda.com, average exchange rate at December 2014). The OOP health expenditure was higher for wealthier households than for the poor, and for urban households than for the rural, although the prevalence of health shocks was quite similar across different wealth quintiles and across rural/urban residential areas. Almost a fifth (17%) of the households in the poorest quintile have no (zero) OOP health expenditure, suggesting that they may have forgone healthcare services due to limited access or affordability.

**Table 3:** Household expenditure (in thousands of L$), reporting of illness and utilization measures

	Total Expenditure	Non-food Expenditure	OOP Health Expenditure	Prevalence of health shocks	Utilization, inpatient
Full Sample	217.8	87.4	2.8	19%	20%
Quintile					
Poorest (1)	107.8	26.6	1.9	16%	23%
2	168.6	46.7	1.9	20%	24%
3	229.9	78.4	3.1	21%	21%
4	310.5	119.0	3.0	19%	21%
Wealthiest (5)	574.4	287.3	3.5	17%	16%
Residence area					
Urban	248.3	111.7	3.2	19%	22%
Rural	171.2	50.2	2.1	18%	18%

Source: Liberia Household income and expenditure survey, 2014. Average exchange rate from Oanda.com, December 2014

### CHE incidence, intensity, equity

CHE incidence and intensity are shown in Table 4. CHE was experienced by between 0.4% (95% CI: 0.2–0.6%) and 2.1% (95% CI: 1.7–2.5%) of the households in Liberia, depending on the threshold used. The intensity of CHE ranged from 14.4% (95% CI: 7.4–21.4%) to 44.6% (95% CI: 24.2–65.0%), depending on the threshold used. In other words, households incurring CHE have incurred, on average, expenditures of 14.4% (95% CI: 7.4–21.4%) of total household expenditure over and above their average household expenditure.

**Table 4:** CHE Incidence and Intensity, by different thresholds

Threshold	As a share of total expenditure		As a share of capacity to pay (non-food expenditure)	
	10%	25%	30%	40%
CHE Incidence (95% CI) [HH count]	1.7% (1.3–2.2%) [70]	0.4% (0.2–0.6%) [19]	2.1% (1.7–2.5%) [102]	1.4% (1.0–1.7%) [69]
CHE Intensity (95% CI)	14.4% (7.4–21.4%)	34.0% (14.5–53.6%)	37.4% (22.7–52.0%)	44.6% (24.2–65.0%)
Concentration Index	−0.03	− 0.44^b^	−0.24^c^	− 0.33^c^
Weighted CHE Incidence	1.8%	0.5%	2.6%	1.8%

^a^ = significant at the 0.05 level, ^b^ = significant at the 0.01 level, ^c^ = significant at the 0.001 level

The concentration indices were negative across all thresholds considered, and only the concentration index calculated at the 10% threshold of total expenditure was not significant against the null hypothesis that CHE is equal across quintiles. As expected, the weighted CHE incidence, resulting from applying the concentration index weighting to the CHE incidence, was higher than the crude/unweighted CHE incidence; implying that CHE was concentrated among poorer households.

**Fig. 2:**
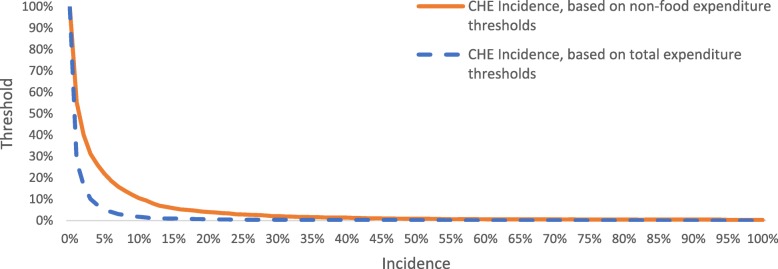
Catastrophic Health Expenditure (CHE) incidence depending on different expenditure thresholds

CHE incidence sensitivity analyses are presented in Fig. 2 and show that CHE incidence was higher when using a non-food expenditure threshold.

### Impoverishment effect of OOP health expenditure

The percentage of households living below poverty line in Liberia before and after considering OOP health expenditures is presented in Table 5. The table shows that 53.6% of sampled households were already living below the overall poverty line (44.1% below the food poverty line) before considering OOP health expenditures. After considering OOP health expenditures, the incidence of sampled households living below the overall poverty line increases by 0.6% (an increase of 1.6% using the food poverty line).

**Table 5:** Impoverishing effects of OOP health expenditure

Poverty Line	% sample households below poverty line		Absolute change [(2)–(1)]	Relative change [((2)/(1))-1]
	Gross of OOP Health Expenditure (1)	Net of OOP Health Expenditure (2)		
Overall poverty line	53.6%	54.2%	0.6%	1.1%
Food poverty line	44.1%	45.7%	1.6%	3.6%

### HFH incidence

**Fig. 3:**
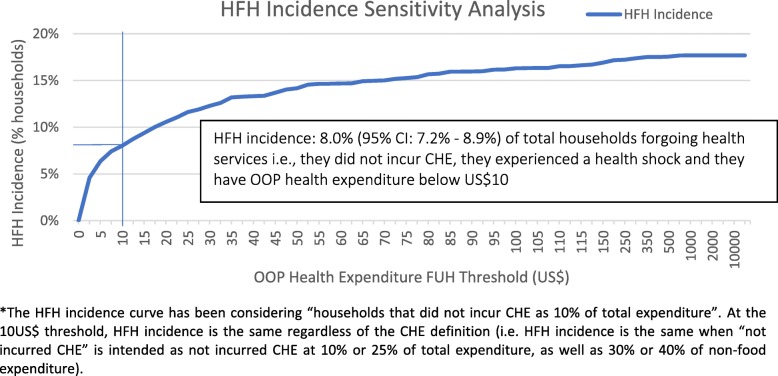
Households forgoing health services (HFH) incidence sensitivity analysis. *The HFH incidence curve has been considering “households that did not incur CHE as 10% of total expenditure”. At the 10US$ threshold, HFH incidence is the same regardless of the CHE definition (i.e. HFH incidence is the same when “not incurred CHE” is intended as not incurred CHE at 10% or 25% of total expenditure, as well as 30% or 40% of non-food expenditure)

The measure of HFH incidence suggests that many households (HFH incidence: 8.0, 95% CI: 7.2–8.9%) have forgone healthcare services needed due to a health shock (i.e., not incurred CHE, experienced a health shock *and* spent less than US$10 in healthcare costs) and therefore are not financially protected. HFH incidence sensitivity analysis (Fig. 3) shows that even more households (towards a maximum of ~ 18% of total households) have experienced a health shock and have not incurred CHE as the threshold value increases.

## Discussion

This study has measured catastrophic health expenditures in Liberia, how this varies by income group and the extent to which these measures represent financial risk protection in a low-income country. OOPs represent 20% of health expenditures in Liberia [36] and so provide a good indicator of financial health risk protection in a health system where many healthcare services cannot be provided by the public health system free at point of care. The study shows that OOP health expenditures are a significant source of financial risk for Liberian households, especially for poorer households (concentration index: − 0.03 to − 0.44). Depending on the threshold used, 0.4% (95% CI: 0.2–0.6%, 25% total expenditure threshold) to 2.1% (95% CI: 1.7–2.5%, 30% non-food expenditure threshold) of Liberian households incur CHE every year, and 1.6% of Liberian households fall into poverty every year due to OOP health expenditure. These findings are consistent with findings from other developing countries e.g. Malawi, Ghana, Swaziland and Mongolia [3, 8, 16, 18]. The findings also show that CHE intensity in Liberia is generally greater than in other countries [3, 6, 18], and it ranges from 14.4% (95% CI: 7.4–21.4%, 10% total expenditure threshold) to 44.6% (95% CI: 24.2–65.0%, 40% total expenditure threshold) depending on the threshold chosen.

The higher intensity of CHE in Liberia is possibly due to widespread poverty and the more frequent occurrence of CHE in lower socioeconomic quintiles. The difference in CHE between urban and rural is also important to note. Since the prevalence of health shocks is similar in rural and urban areas, OOP health expenditure and utilization of inpatient services should be similar across urban and rural areas but this is not the case. There are lower OOP health expenditures and healthcare utilization rates in rural areas. This suggests that healthcare services in rural areas are inaccessible due to either supply issues (i.e., there are few or no healthcare providers) and/or demand issues (i.e., rural households are poorer and cannot afford to buy healthcare).

The analysis demonstrates that while CHE incidence can “look” low at higher thresholds, it is in fact very high when the threshold is low. To understand this variation better, the CHE incidence curve allowed a comparison of CHE incidence based on total expenditure and non-food expenditure. The higher CHE incidence resulting from using non-food expenditure thresholds suggests that Liberians are not substituting food for healthcare, probably because food consumption is already low (percentage of households below the food poverty line: ~ 45% [15]). However, neither of these analyses can take account of the decision not to incur health expenditures because of financial risk or sub-optimal supply of healthcare services as suggested by the rural-urban differences in CHE. The study proposed a simple method to explore the extent to which this happens in the form of incidence of healthcare forgone (HFH) (experiencing a health shock, having OOP health expenditure that is too low to cover most health conditions (<US$10) while not experiencing CHE).

HFH incidence was over 5 times the incidence of impoverishing expenditure and 3 times the incidence of CHE (at the higher threshold). Given the high rates of poverty in Liberia, the implication is that these households could not afford or access the healthcare services that they need. It raises the question whether CHE is a sufficient measure of financial risk protection where high levels of poverty lead to low uptake of services and whether improved financial risk protection understanding may impact decision making; for example, policy makers may consider actions that address HFH. It is important to remember that those households that forgo healthcare may not spend money on healthcare but the impact of untreated illness may then lead to impoverishment in other ways such as reduction in productivity and/or income.

The study suffers from some limitations, most notably the small sample size of individuals experiencing CHE. For a threshold equal to 25% of total expenditure, the sample i.e., households incurring CHE, is small (19 households). The study addresses this by varying the threshold and the use of the CHE incidence curves which indicate that the incidence rates are still relatively high at the lower threshold level. However, for the measure of intensity the small sample size leads to parameter uncertainty (i.e., low number of households incurring CHE), and for this reason, the confidence intervals for the intensity measure are very large. Other survey related limitations include the 30-day recall period for some of the health expenditures being considered in OOP health expenditures and the fact that other non-financial costs (e.g. time spent to receive healthcare services) have not been considered due to data availability limitations. Since income is annual, annualization was required. It is impossible to know with certainty to what extent such “past 30 days” expenses have been repeated during the full year. However, the impact of this is unknown and could result in an under- or over-estimation of CHE. In addition, this study only included cross-sectional data from HIES 2014. The HIES 2016 data, which is now available [13], would capture the impact of the Ebola virus as well as facilitate sub-group analysis. However, these limitations do not impact the demonstration of the effectiveness of HFH incidence to assess financial risk protection and the analysis of the 2014 survey can be taken as a baseline and indicate areas of further research when assessing financial health risk protection between 2014 and 2016.

The HFH incidence measure is a proposition. HFH could be measured via adding questions to surveys (e.g. living standards surveys) or by using more elaborate needs-based approaches, as proposed in the literature [17, 21]. It should also be noted that this measure does not explore *why* people may not get the healthcare they need, and that the reasons for forgoing healthcare may not be financial (e.g. voluntary refusal to get modern healthcare, not knowing that a treatment exist, impossibility to access treatment or information due to non-financial reasons). It is also important to note that some households may have accessed healthcare without a financial expenditure (e.g. where services have been provided free of charge as a goodwill gesture or via a health program, or where services were paid directly by another household). While a sensitivity analysis was provided, it was not possible to assess this formally. Consequently, the results may represent an over-estimate of actual HFH.

## Conclusion

Financial catastrophe due to OOP health expenditure is a significant problem in Liberia and adversely affects the poorer socio-economic quintiles. Whereas catastrophic health expenditure incidence provides a good measure of the degree to which a population is affected by OOP, incidence curves provide a useful way to compare the impact of different thresholds and different measures of CHE. However, financial risk protection measures are incomplete as they fail to include those households not accessing healthcare due to financial constraints. A simple measure of incidence of households forgoing healthcare shows this is a significant problem in Liberia. The high level of CHE, impoverishing expenditure and HFH support the implementation of health system reforms that are equitable and increase financial risk protection in Liberia, protecting Liberians, especially the poorest, from financial catastrophe and from forgoing healthcare. These findings can also be used as a baseline against which future evaluations of CHE and equity of health policies can be measured. Although more research is needed to better understand the consequences of forgoing healthcare and improve the methodology for measuring its incidence, the measure of HFH incidence proposed in this paper provides a simple and flexible method to demonstrate its importance.

## Data Availability

The datasets used is available publicly on World Bank Microdata portal at http://microdata.worldbank.org/index.php/catalog/2563. The dataset including analyses is available from the corresponding author on reasonable request.

## Appendix 1

### Catastrophic health expenditure incidence methods

If we let *T* be OOP health expenditure and *x* be the household resources of a given household, *i*, then headcount of CHE in the form of the dummy variable *HC* is calculated as follows:

$$ H{C}_j=1\  if\ \left(\frac{T_i}{x_i}\right)-{z}_j>0,0\  otherwise $$

where, *z* is the threshold that can assume only two *j* values: 10% or 25% of *x*_*i*_ when *x* is total expenditure; and, 30 and 40% when *x* is capacity to pay. CHE incidence is then calculated as:

$$ {\overline{HC}}_j=\frac{1}{N}\sum \limits_{i=1}^N{HC}_{ij} $$

## Appendix 2

### Catastrophic health expenditure intensity methods

*T* be OOP health expenditure and *x* be the household resources of a given household, *i*, then intensity of CHE takes the form of a quantitative variable *O* and is calculated as follows:

$$ {O}_{ix}=\left(\frac{T_i}{x_i}\right)-{z}_j $$

where, *z* is the threshold that can assume only two *j* values: 10% or 25% of *x*_*i*_ when *x* is total expenditure; and, 30 and 40% when *x* is capacity to pay.

The average intensity for the population experiencing CHE (i.e., positive mean overshoot), $\overline{O}$, is as follows:

$$ {\overline{O}}_{jx}=\frac{\sum \limits_{i=1}^N{O}_{ijx}}{\sum \limits_{i=1}^N{HC}_{ijx}} $$

## Appendix 3

### Equity and CHE methods: concentration index

A “rank-weighted CHE incidence” was estimated to take account of the distribution of CHE incidence across different socio-economic quintiles. In mathematical terms, the rank-weighted CHE incidence $\overline{WHC}$ was calculated using concentration index *C*_*HC*_ and CHE incidence $\overline{HC}$ as follows:

$$ \overline{WHC}=\overline{HC}\ast \left(1-{C}_{HC}\right) $$

Where, the concentration index headcount is

$$ {C}_{HC}=\frac{2}{\mu_{HC}}\mathit{\operatorname{cov}}\left( HC,\mathit{\operatorname{rank}}\right) $$

And (− 1 < *C*_*HC*_ < + 1).

The concentration index provides a distribution of the socioeconomic status across the sampled households [2, 6, 8]. The index *C*_*HC*_ was calculated using the conindex Stata command [40] through which households were allocated weights according to their rank in the socio-economic distribution. The concentration index distribution gives a weight of 2 (the poorest households) and 0 (the richest) [18, 29]: the result is that *C*_*HC*_ is equal to zero when CHE incidence is distributed perfectly equally (with regards to income), negative if CHE is concentrated among poor households and positive if CHE is concentrated among rich households. Normalised indices [39] for binary variables have been calculated too to check for any difference versus the standard concentration index. Since HIES 2014 sampling methods are probabilistic, sampling weights have been applied using the pweight option.

## Appendix 4

### Impoverishing effect methods

Household poverty line for each household has been measured in the following way: if household i has n individual household members j, and each individual household member has an adult equivalence score *AE*_*j*_, then each household i poverty line *HHPL*_*i*_ is defined as:

$$ {HHPL}_i=\left(\sum \limits_{j=1}^n{AE}_j\ast {Individual}_j\right)\ast 65438\ L\$ $$

where 65,438 L$ is the Liberian poverty line per each adult equivalence, as per the HIES 2014 methodological appendix [14].

A dummy variable *p* − *pre*_*i*_ takes the value 1 when household *i* total consumption expenditure *x*_*i*_ is below its poverty line *HHPL*_*i*_, and 0, otherwise, then the incidence of poverty prior to OOP is:

$$ \overline{p- pre}=\frac{1}{N}\sum \limits_{i=1}^Np-{pre}_i $$

where *p* − *pre*_*i*_ = 1 *ifx*_*i*_ < *HHPL*_*i*_, 0 *otherwise*

It is implied in this *p* − *pre*_*i*_ formula that OOP health expenditure do NOT affect the household income as it is compared to the poverty line.

A dummy variable *p* − *post*_*i*_ takes the value 1 when household *i* total consumption expenditure *x*_*i*_ minus OOP health expenditure *T*_*i*_ is below the poverty line *HHPL*_*i*_, and 0, otherwise:

$$ \overline{p- post}=\frac{1}{N}\sum \limits_{i=1}^Np-{post}_i $$

where *p* − *post*_*i*_ = 1 *if*(*x*_*i*_ − *T*_*i*_) < *HHPL*_*i*_, 0 *otherwise* [7, 17, 29].

In this *p* − *post*_*i*_ formula, the total consumption expenditure *X*_*i*_ of household i is discounted by the OOP health expenditure *T*_*i*_. The impoverishing effect of OOP health expenditure is the difference between poverty incidence before OOP health expenditure ($\overline{p- pre}$) and poverty incidence after OOP health expenditure ($\overline{p- post}$).

## Appendix 5

### Households forgoing healthcare methods

Let HC be the binary variable representing whether household i incurred CHE, Health Shock a binary variable representing whether household i reported a health shock or not, and *T* be household i OOP health expenditure, then HFH is a binary variable defined as:

$$ {HFH}_{ij}=1\  if\  HC=0, Health\ Shock=1\  and\ {T}_i< US\$10;0\  otherwise $$

The incidence of HFH at the population level is estimated as:

$$ {\overline{HFH}}_{jx}=\frac{1}{N}\sum \limits_{i=1}^N{HFH}_{ijx} $$

using total expenditure x as CHE HC denominator, and different CHE thresholds j.
