# Correction to: Households forgoing healthcare as a measure of financial risk protection: an application to Liberia

**DOI:** 10.1186/s12939-020-1132-x

**Published:** 2020-02-05

**Authors:** Jacopo Gabani, Lorna Guinness

**Affiliations:** 1 Centre for Health Economics, University of York, York, UK; 2 London School of Hygiene & Tropical Medicine, London, UK

**Correction to: Int J Equity Health**

**https://doi.org/10.1186/s12939-019-1095-y**

We have been notified after publication of the article [1], that the sentence of the Results part of the Abstract text is mentioned with the mistake. Currently the sentence is: c approximately 4 times higher than CHE (8.0, 95% CI, 7.2– 8.9%). It should, however, read as follows: HFH incidence is approximately 4 times higher than CHE (8.0, 95% CI, 7.2–8.9%). The publisher apologizes to the authors and readers for the error & inconvenience. The original article has been corrected.
